# Recurrent discharges of non-petroleum substances from chemical tankers in Swedish marine Natura 2000 sites are against the aims of EU Directives

**DOI:** 10.1007/s13280-024-02103-7

**Published:** 2024-11-19

**Authors:** Kjell Larsson, Ulrica Carlson, Erik Stålnacke

**Affiliations:** 1 Kalmar Maritime Academy, Linnaeus University, 391 82 Kalmar, Sweden; 2 Swedish Coast Guard, Box 536, 371 23 Karlskrona, Sweden

**Keywords:** Baltic Sea, Birds directive, Habitats directive, MARPOL annex II, Shipping, Tank washing

## Abstract

The transport of non-petroleum substances such as vegetable oils, other bio-based oils and their refined products by chemical tankers is increasing worldwide. The majority of the non-petroleum substances carried by chemical tankers will have detrimental effects on the marine environment if accidentally spilled or discharged during tank washing procedures. Swedish Coast Guard aircrafts detected 233 discharges of floating non-petroleum substances in the Swedish territorial sea and Swedish Exclusive Economic Zone (EEZ) between 2020 and 2023. The majority of the discharges, 84%, were detected in the EEZ. About 14% of the discharges were detected within protected marine Natura 2000 sites. Together, the detected discharges covered 1071 km^2^ of sea surface. Discharges in marine Natura 2000 sites covered 228 km^2^. We conclude that the regulations in the IMO MARPOL Annex II convention are not strict enough to meet the objectives of EU nature legislation regarding protection of sensitive seas areas.

## Introduction

The production, consumption and sea transport of non-petroleum substances such as vegetable oils, other bio-based oils and their refined products are increasing worldwide (OECD/FAO 2021a, 2021b; Chemanalyst 2024; Churchill et al. 2024). Vegetable oils, for example, palm oil, rapeseed oil and soybean oil, as well as substances such as tall oil, a renewable residual product from pulp mills, are used as raw material in the manufacturing industry and in some cases as food. These substances and various fats are also in increasing amounts refined to biofuel, that is, to Fatty Acid Methyl Esters (FAME) and Hydrotreated Vegetable Oil (HVO). Because of the large volumes involved, the substances are transported worldwide at sea by specialized chemical tankers or by combined chemical/oil product tankers.

The majority of the non-petroleum substances carried by chemical tankers, including vegetable oils, other bio-based oils and biofuels, will have detrimental effects on marine species and habitats if accidentally spilled or intentionally discharged to the sea (Hollebone et al. 2008; Fingas 2015; GESAMP 2019; Tamothran et al. 2022). To reduce the risk of negative effects of pollution, the International Maritime Organization (IMO) has created a regulatory framework for the sea transport of chemicals in bulk, including regulations for the control of operational discharges of residues of the carried chemicals. The main regulatory texts concerning non-petroleum substances are the MARPOL convention Annex II “Regulations for the control of pollution by noxious substances in bulk”, and the IBC Code “International Code for the Construction and Equipment of Ships Carrying Dangerous Chemicals in Bulk” (IMO 2020, 2022). Substances covered by the regulations in MARPOL Annex II and the IBC Code include biofuels, vegetable oils, acids, bases, alcohols, and hundreds of other substances listed in chapter 17 of the IBC Code, but not petroleum oils. The sea transport of crude petroleum oil and petroleum oil products is regulated separately in the MARPOL convention Annex I “Regulations for the prevention of pollution by oil”. Henceforth, in the current study, the focus is on legal and illegal discharges of a subset of substances, namely vegetable oils, other bio-based oils and biofuels, that is, non-petroleum substances that when floating can be detected from surveillance aircrafts (Bonn Agreement 2011).

Accidents leading to very large discharges from chemical tankers sometimes occur, but a more common pathway by which chemicals from the ships’ cargo tanks enter the marine environment is through legal and illegal tank washings (Bucas and Saliot 2002; Häkkinen and Posti 2014; Şanlier 2018; Lunde-Hermansson and Hassellöv 2022). Because chemical tankers after unloading of a cargo tank usually will load a different non-compatible substance in the same tank, tank washing operations are needed for safety and commercial reasons. How and when tank washing operations shall be performed, and if the contaminated wash water must be discharged to a reception facility or could be released back to sea is dependent on the type of substance transported. Chemicals carried by chemical tankers are divided into four categories X, Y, Z and OS (IMO 2022). Category X substances are deemed to present a major hazard, Y substances a hazard, and Z substances a minor hazard to either marine resources or human health. The Category OS (Other Substances) are, at present, considered to present no harm (IMO 2022).

Before any tank washing operations or discharge procedures are carried out, the tank shall be emptied at the port of unloading to the maximum extent according to the ships’ procedures and arrangements manual (P&A Manual). A tank from which a category X substance has been unloaded must be prewashed before the ship leaves the port and the resulting wash water and residues must be discharged to a reception facility. The same rules apply when a tank has contained Y substances that are highly viscous, solidifying or persistent floaters (IMO 2020, 2022). When chemical tankers have transported and unloaded other harmful substances in the Y and Z categories a prewash of the tanks is not mandatory. Such tanks, and previously prewashed tanks, can later be washed legally at sea given that the ship is proceeding en route at a speed of at least 7 knots, the discharge is made below the waterline and is made at a distance not less than 12 nautical miles from nearest land, and in waters that is not less than 25 m deep (IMO 2022). For ships built after 1 January 2007 a maximum of 75 L of residues of category X, Y, or Z substances per cargo tank may be discharged to the sea. Older ships may discharge 150 L of residues of X and Y substances and 350 L of Z substances per cargo tank. Because chemical tankers have several cargo tanks, the total volume of residues that may be legally discharged to the sea after each unloading may amount some hundred litres.

It should be noted that MARPOL Annex II lack stricter regulations for discharges in sensitive and protected marine areas. The only defined Special Area under the Annex II, with a higher level of protection, is the Antarctic area south of latitude 60º S, where discharges into the sea of noxious liquid substances or mixtures containing such substances is prohibited (IMO 2022). This contrasts to other international environmental conventions and regulations that often recommend special and stricter protection of the most sensitive areas and sites. At the global level, the parties to the UN Convention on Biological Diversity (CBD) have adopted a goal to protect sites covering at least 30% of marine areas by 2030 in order to conserve marine biodiversity and ecosystems, including to reduce negative impacts of pollution (CBD 2022). At the EU level, the designation of a network of protected Natura 2000 sites under the Habitats and Birds Directives is an important tool, and a legal base, to protect sensitive species and habitats and reduce pollution in the territorial seas and Exclusive Economic Zones (EEZ) of EU member states.

In this study, we present data on detected discharges of floating non-petroleum substances from ships in the Swedish territorial sea and Swedish EEZ between 2020 and 2023. We analyse the occurrence of discharges in protected marine Natura 2000 sites and point out potential effects on marine species and habitats. We conclude that the regulations in the IMO MARPOL Annex II convention at present are not strict enough to meet the objectives of EU nature legislation regarding protection of sensitive seas areas.

## Materials and methods

### Aerial surveillance

Discharges of tank washing residues other than petroleum oil were detected by the Swedish Coast Guard between 2020 and 2023 when performing regular aerial surveillance of the Swedish territorial sea and the Swedish EEZ. Discharges were detected visually or by sensors like Side Looking Airborne Radar (SLAR). Some discharges within the Swedish EEZ were also reported by Danish, German and Finnish surveillance aircrafts in line with the agreement to cooperate on aerial surveillance in the Baltic Sea (HELCOM 2024). Indications of discharges were also obtained from analyses of satellite images received from the European Maritime Safety Agency. These indications were often checked, if possible during the same or consecutive day, by the surveillance flights. Only discharges that were judged to originate from ships at sea were included in the further analyses. Thus, discharges in or close to harbours, in inland waters, and discharges from land sources were not included.

### Distinguishing between non-petroleum substances and petroleum oils

Discharges of floating non-petroleum substances originating from tank washing operations may appear in many different ways, but they do not appear in the same way as petroleum oil. The appearances of discharges of petroleum oil on water have been described in detail in the Bonn Agreement Oil Appearance Code (BAOAC) (Bonn Agreement 2024). A method to estimate the thickness of the petroleum oil layer, based on the different appearances, has also been developed. Unfortunately, no such detailed method has hitherto been developed for non-petroleum substances. However, it is still possible to distinguish discharges of non-petroleum substances from petroleum oils according to their deviant colours and appearance.

Discharges of non-petroleum substances may appear colourless but also clearly black, brown, white, amber, yellow, etc. depending on the type of substance. The discharges often consist of lumps of substances of various size. These lumps may slowly dissolve and emit hardly visible thin shiny slicks that resemble a calm sea. Discharges of vegetable oils are also usually not spreading out as fast as petroleum oils from the discharging ship. More details about the different appearances of non-petroleum substances and petroleum oils on water can be found in the Bonn Agreement Oil Appearance Code Photo Atlas (Bonn Agreement 2011). Thus, although it is in most cases possible to discriminate between discharges of non-petroleum substances and petroleum oils from the surveillance aircraft it is usually not possible, from visible cues only, to obtain more detailed information on the non-petroleum substance, for example, to discriminate between various types of vegetable oils and other bio-based oils.

### Information on the discharged non-petroleum substances

When an ongoing discharge from a ship was detected, information on the discharged substance could be directly obtained from the radio conversation between the Swedish Coast Guard and the crew of the discharging ship. The Swedish Coast Guard regularly also asked for hard copies of the cargo record book from the ship. The control of cargo record books is a common method to obtain information about the substances carried and to confirm compliance to MARPOL regulations. Information on the substances carried was also in some cases obtained later from personnel at ports of unloading. If a discharging chemical tanker during ongoing tank cleaning was en route more than 12 nautical miles from land and the surveillance aircraft, based on the information at hand, did not suspect non-compliance to MARPOL regulations, no further actions was taken by the coast guard.

When possible and when discharges of non-petroleum substances were detected less than 12 nautical miles from land, and, hence, were illegal, the detected floating slicks were for evidentiary purposes sampled from the sea surface to determine the type of substance discharged. Sampling was performed either by dropping a sampling buoy from an aircraft or by taking samples from a boat. The sampling buoy was later retrieved from a boat. A special cloth was used to collect and conserve the substance for further analyses at a laboratory.

### Detection possibilities depend on external factors

Although aerial surveillance was performed regularly in large parts of the Swedish territorial sea and EEZ, certainly not all discharges from ships were detected. Strong winds, which are more common in winter, create rough waves and a rough sea state, which in turn will shatter slicks on the sea surface and mix floating substances into the water column, and, hence, make detections from aircrafts more difficult. How long time discharges are visible on the sea surface depends also on the type and quantity of cargo residues that have been discharged. The experience of coast guard crews is that detected discharges of non-petroleum substances might be visible on the sea surface during less than a day up to several days.

The Swedish Coast Guard’s surveillance effort is mainly directed to the busiest ship routes where many discharges can be expected. However, the behaviour of chemical tankers that are planning a tank washing operation may sometimes deviate from normal navigation practice along ship routes (Riksrevisionen 2024). Because of the varying surveillance effort, traffic intensity and varying weather it is difficult to make any detailed estimate of the number and geographic distribution of undetected discharges during the study period, but it is at least reasonable to assume that the number of undetected discharges is considerably higher in winters.

### Size and volume of discharges

At present, no standardized quality assured method has been developed to use remote sensing equipment to estimate the thickness of films of floating non-petroleum substances. Hence, it was not possible to directly estimate the volume of the detected discharges from the information obtained during aerial surveillance. The size of the discharges are therefore expressed as the area (in km^2^) of the sea surface covered when detected.

## Results

**Fig. 1:**
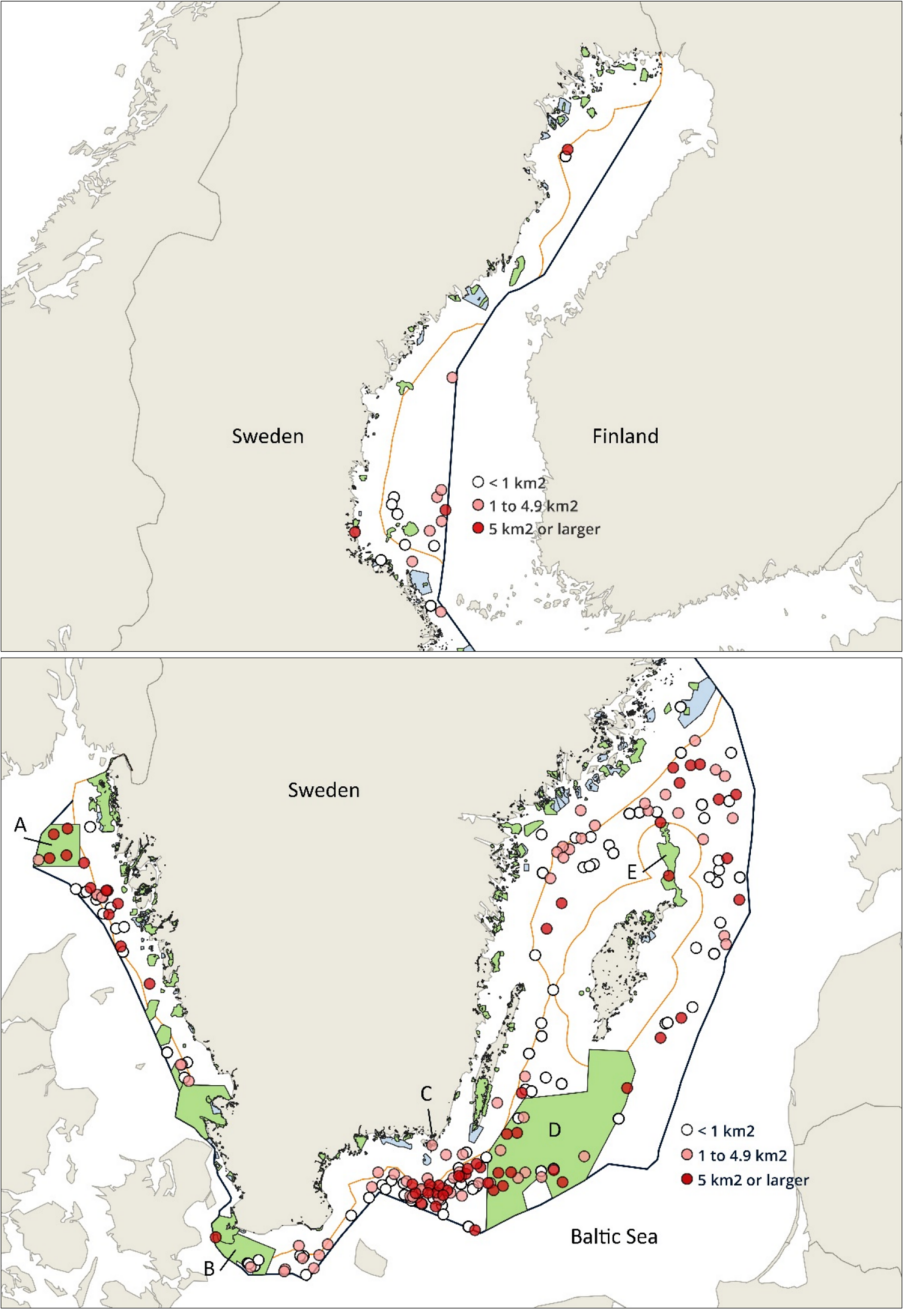
Location of detected discharges of non-petroleum substances in the northern part (above) and southern part (below) of the Swedish territorial sea and Swedish EEZ between 2020 and 2023. Size of discharges are indicated by the colour of dots. Green areas show coastal and marine Natura 2000 sites. Blue areas show coastal and marine nature reserves outside Natura 2000 sites. Yellow and black lines show the borders of the Swedish territorial sea and the Swedish EEZ, respectively. Note the different scales of the two maps. Borders of Natura 2000 sites and nature reserves were obtained from Swedish Environmental Protection Agency (2024). Letters A to E refer to the Natura 2000 sites Bratten, Sydvästskånes utsjövatten, Torhamn-Hästholmen, Hoburgs bank och Midsjöbankarna, and Gotska Sandön-Salvorev, respectively

The surveillance aircrafts detected 233 discharges of non-petroleum substances between 2020 and 2023 in the Swedish territorial sea and in the Swedish EEZ (Fig. 1). The majority of the discharges were detected in the Baltic Proper, Kattegatt and Skagerrak, that is, in areas with very intensive ship traffic. Especially large number of discharges were detected in the sea between the southeastern tip of Sweden and the Danish island of Bornholm. Considerably fewer discharges were detected in the Gulf of Bothnia.

About 15% of the discharges (n = 34) were detected within marine protected areas. Five different marine Natura 2000 sites and one marine nature reserve, named Svenska Högarna, not being a Natura 2000 site were affected (Fig. 1, Table 1). Three of the marine Natura 2000 sites, Bratten, Sydvästskånes utsjövatten and Gotska Sandön-Salvorev, were designated under the Habitats Directive (Special Areas of Conservation, SACs), one site, Torhamn-Hästholmen, under the Birds Directive (Special Protection Areas, SPAs) and one site, Hoburgs bank och Midsjöbankarna, under both directives (SAC and SPA). Another eight discharges were detected within less than 5 km from boundaries of marine protected areas.

**Table 1:** Number of detected discharges of non-petroleum substances in Swedish territorial sea, Swedish EEZ and in marine protected areas

Year	Total number of detected discharges	In Swedish territorial sea	Per cent in Swedish territorial sea	In Swedish EEZ	Per cent in Swedish EEZ	Within Natura 2000 sites	Within nature reserves outside Natura 2000 sites	Per cent within marine protected areas
2020	53	7	13.2	46	86.8	11	1	22.6
2021	57	12	21.1	45	78.9	5	0	8.8
2022	54	5	9.3	49	90.7	6	0	11.1
2023	69	13	18.8	56	81.2	11	0	15.9
Total	233	37	15.9	196	84.1	33	1	14.6

The majority of the discharges, about 84% (n = 196), were detected in the Swedish EEZ. About 16% (n = 37) of the discharges were detected in the Swedish territorial sea (Tables 1 and 2). Three discharges were detected in the Swedish EEZ but less than 12 nautical miles from land. They were detected at sites at the Swedish south coast where the border of the Swedish territorial sea does not extend to 12 nautical miles due to special international agreements. Of the 23 discharges that were detected very close to the territorial border, that is, within 2 km of either side, 17 discharges were detected just outside the border, and six discharges were detected inside the border in the territorial sea.

**Table 2:** Sea surface covered (in km^2^) of detected discharges of non-petroleum substances in Swedish territorial sea, Swedish EEZ and in marine protected areas

Year	Total surface covered	In Swedish territorial sea	Per cent in Swedish territorial sea	In Swedish EEZ	Per cent in Swedish EEZ	Within Natura 2000 sites	Within nature reserves outside Natura 2000 sites	Per cent within marine protected areas
2020	309.45	13.77	4.4	295.68	95.6	50.88	0.02	16.4
2021	165.16	29.67	18.0	135.50	82.0	55.68	0	33.7
2022	256.02	21.60	8.4	234.42	91.6	33.66	0	13.1
2023	340.05	87.20	25.6	252.85	74.4	87.74	0	25.8
Total	1070.68	152.23	14.2	918.45	85.8	227.96	0.02	21.3

### Substances discharged

In 31 cases it was possible by various means, including communication with the discharging ships, control of cargo record books and in a few cases laboratory analyses, to verify the type of substance discharged. The identified substances were Fatty Acid Methyl Esters (FAME), including Rapeseed Fatty Acid Methyl Esters (RME) (10 cases), tall oil, including tall oil crude and tall oil pitch (9 cases), palm oil (6 cases), soybean oil (1 case), rapeseed oil (1 case), Mixed Fatty Acids (MFA) (2 cases) and calcium carbonate slurry (2 cases). It was not possible to verify if detected discharges of FAME to some percentage were blends with petroleum diesel. The categorisation of the verified substances according to the IBC Code is shown in Table 3.

**Table 3:** Properties of discharged non-petroleum substances detected in the Swedish territorial sea or EEZ. The letter in the pollution category refers to the assigned substance category under MARPOL Annex II. In the hazards column the letter P means that the substance is included in the IBC Code because of its pollution hazards, and S means that the substance is included because of its safety hazards. A prewash of cargo tanks after unloading is required when transported substances are categorized in the X category, or in the Y category when also categorized as high-viscocity or solidifying substances, or as persistent floaters. Data is obtained from Resolution MEPC.318(74) (IMO 2019). *When biofuel blends are containing 75% or more of petroleum oil, the biofuel blend is subject to regulations in Annex I of MARPOL. Discharges of such blends and other petroleum oils are prohibited in the Baltic Sea and North Sea

Substance	Pollution category	Hazards	Persistent floaters	Prewash required
Fatty Acid Methyl Esters (FAME)	Y	P and		
Rapeseed Fatty Acid Methyl Esters (RME)	Y	P and S		
Bio-fuel blends of Diesel/gas oil and FAME (> 25% but < 99% by volume)*	X	P and S		Yes
Bio-fuel blends of Diesel/gas oil and vegetable oil (> 25% but < 99% by volume)*	X	P and S		Yes
Tall oil crude	Y	P and S		
Tall oil pitch	Y	P		
Palm oil	Y	P	Yes	Yes
Soybean oil	Y	P and S	Yes	Yes
Rapeseed oil	Y	P	Yes	Yes
Mixed Fatty Acids (MFA) see below				
Fatty Acid (saturated C13 +)	Y	P and S		
Fatty Acids, (C8-C10)	Y	P and S		
Fatty Acids, (C12 +)	Y	P and S	Yes	Yes
Fatty Acids, (C16 +)	Y	P		
Fatty acids, essentially linear (C6-C18) 2-ethylhexyl ester	Y	P and S		
Calcium carbonate slurry	OS			

In the other 202 cases the experienced coast guard aircraft crews made the judgement during the aerial surveillance that the observed floating substances were non-petroleum substances, thus, neither petroleum oil nor discharges of sewage, litter or substances of natural origin, for example floating algae belts. The judgements were based on long-term previous experience of the appearance of spills of different substances as described in the methods section.

### Size of discharges

**Fig. 2:**
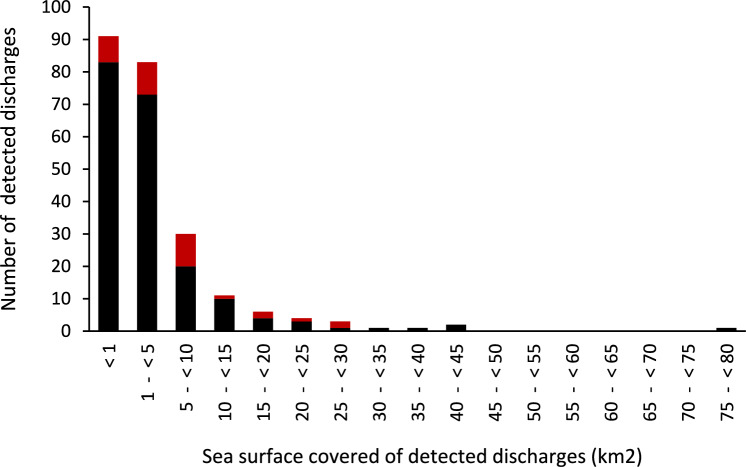
Size distribution of detected discharges. Red columns show the number and size of discharges in marine protected areas

The size of the 233 detected floating discharges, measured as the sea surface covered, varied between 0.01 km^2^ and 78 km^2^. About 61% of the discharges covered a sea surface of 1 km^2^ or more and about 25% of the discharges covered a sea surface of 5 km^2^ or more (Figs. 1 and 2). The total size of the 59 largest discharges, which each covered 5 km^2^ or more, added up to about 79% of the total sea surface covered by all 233 detected discharges. The total size of the 34 discharges detected within marine protected areas added up to about 21% of the total sea surface covered by all discharges (Table 2).

## Discussion

### Legal vs illegal discharges

According to MARPOL Annex II (IMO 2022) and to Swedish national regulations (SFS 1980:789), all discharges less than 12 nautical miles from nearest land of substances in the X, Y and Z categories are prohibited. Thus, the 37 discharges that were detected in the Swedish territorial sea, plus three discharges detected in the Swedish EEZ but less than 12 nautical miles from land, were clearly the result of illegal acts. Of the discharges that were detected very close to the border of the territorial sea most were detected just outside the border. This indicates that many ships adjusted the discharging procedure to the location of the territorial border and to the discharge regulations in force in the territorial sea and EEZ, respectively. The legal mandate of the Swedish Coast Guard to act against ships that are suspected to have performed illegal discharges, either because of having discharged at sites less than 12 nautical miles from land, or discharged too large volumes, is stronger in the territorial sea than in the EEZ (SFS 1980:424).

The MARPOL convention Annex II have defined how many litres of residues of category X, Y, or Z substances that may be discharged legally to the sea after unloading. For ships built after 1 January 2007 there is a limit of maximum 75 L per cargo tank. However, it is at present difficult to obtain juridical proof that the volume of a specific discharge detected more than 12 nautical miles from land is larger than the allowed limit. The area covered by floating discharges can be measured with accuracy from surveillance aircrafts. But more analyses and experiments are needed for a range of non-petroleum substances to describe the relationships between the appearances of discharges and the thickness of the films on the surface. The thickness and area covered are the variables needed to obtain a more precise estimate the discharged volume.

However, from previous experiences of the behaviour of vegetable oil films on water (Bonn Agreement 2011), it can be concluded that it is extremely unlikely that a visible floating discharge of vegetable oils that cover a sea surface of more than 10 km2 could be a result of a legal tank washing. Films of petroleum oils that are thinner than 40 nm are invisible (Bonn Agreement 2024). Assuming a similar visibility limit for films of vegetable oils and other bio-based oils, a visible floating discharge covering 10 km^2^ must at least consist of 400 L. Furthermore, because vegetable oil is not spreading out as much as petroleum oil (Fingas 2015; Bonn Agreement 2011), that is, the films would generally be thicker, a visible discharge that cover 10 km^2^ will most likely consist of far more than 400 L.

### Effects of discharged substances on species and habitats

Several previous studies have shown that discharges of non-petroleum substances into the sea can be harmful to a whole range of marine species and habitats. The pathways by which organisms and habitats can be affected by the specific substances varies though. In the current study, it was possible to determine the discharged substance in 31 cases. Most common were discharges of FAME including RME, various forms of tall oil, and vegetable oils such as palm oil, soybean oil and rapeseed oil. Although the exact substance could not be verified for the other 202 detected discharges, we argue, based on the general appearance of the discharges, that it is reasonable to assume that they to a large extent also consisted of the above-mentioned substances.

Vegetable oils and other non-petroleum substances may coat bird’s plumages similarly to petroleum oils and cause loss of insulating capacity, loss of buoyance and subsequent death (McKelvey et al. 1980; Mudge 1995; Bucas and Saliot 2002; Fingas 2015; Tamothran et al. 2022). Even spills only 40 nm thin may disrupt bird feathers and make them non-waterproof (Morandin and O'Hara 2014). Birds may also be negatively affected by ingesting various harmful oily substances while preening damaged feathers (Crump-Wiesner and Jennings 1975).

In the current study, several large discharges of non-petroleum substances were detected in the Natura 2000 site Hoburgs bank and Midsjöbankarna, which is a globally important wintering site where several hundred thousand seaducks and thousands of other seabirds winter (Durinck et al. 1994; Skov et al. 2011; Nilsson 2016). Earlier, between 1996 and 2004, tens of thousands of long-tailed ducks (*Clangula hyemalis*) were killed each year at that site due to recurrent discharges of petroleum oils (Larsson and Tydén 2005). Large, or recurrent small, discharges of non-petroleum substances in this Natura 2000 site can have similar negative effects. Also other sea areas that were affected by the detected discharges in this study, including the Natura 2000 site Gotska Sandön-Salvorev, host large numbers of wintering seaducks and other seabirds (Skov et al. 2011; Nilsson 2016).

Discharges of vegetable oils have also been shown to negatively affect benthic fauna through smothering, oxygen depletion and toxic effects. The effects of rapeseed, linseed, olive and sunflower oils on the performance of blue mussels (*Mytilus edulis*) were investigated by Salgado (1995). All the vegetable oils studied were found to have an inhibitory effect on the growth of mussels. Vegetable oils also caused a change in the fatty acid composition of the mussels and increased mortality rates.

Discharges of vegetable oils may polymerize in the water column, on water surfaces and on sediments, which reduce the degradation rate of the oils (Mudge 1995; Tamothran et al. 2022). Polymerized vegetable oils may form an impermeable cap on the water surface or over the bottom and make the water and sediment below the cap oxygen free due to reduced oxygen diffusion. Oxygen levels may also decrease when vegetable oils are degraded by oxygen consuming bacteria. A decrease in available oxygen in water and sediments can change the species diversity in the affected area (Mudge 1995).

Toxic effects of vegetable oils on crustaceans and other organisms have been found in several studies (Tamothran et al. 2022). The toxicity of vegetable oil spills is thought to mainly arise because of the toxic effects of intermediate products formed during biodegradation, such as long- and medium-chain free fatty acids (Salam et al. 2012; Tamothran et al. 2022). The toxic effects are assumed to be transient effects as the vegetable oil and intermediate products eventually will be degraded by bacteria.

The toxicity of biodiesel (FAME) to aquatic organisms has mainly been studied under laboratory conditions, and biodiesel has generally been found to be less toxic than petroleum diesel partly because biodiesel lacks acutely toxic aromatic and polycyclic aromatic components and volatiles (Hollebone et al. 2008; ITOPF 2024; Xin et al. 2024). However, Xin et al. (2024) found that biodiesel in water was extremely toxic to *Vibrio fischeri* in Microtox tests. Alkylated PAHs and unquantified free fatty acids in biodiesel were probably the cause of their toxicity to *Vibrio fischeri* bacteria. Biodiesel is often blended with petroleum diesel and blends with low proportions of biodiesel, up to 20% biodiesel (B20), have a toxicity similar to petroleum diesel. It has been found that different biodiesels behave differently when spilled in water and have different toxicities, possibly due to differences in feedstock or additives (Hollebone et al. 2008; Xin et al. 2024). Biodiesels generally degrade faster than petroleum diesel (Wilms et al. 2023). Discharges of biodiesel may as petroleum diesel kill marine organisms due to smothering (Hollebone et al. 2008).

Thus, given the results of previous studies, there are compelling reasons to conclude that discharges of non-petroleum substances from chemical tankers can have significant negative effects, not only on coastal and marine birds, but also on pelagic and benthic marine species and habitats. However, to evaluate the specific effects the discharges detected in the current study have had on pelagic and benthic marine species and habitats are not possible given the lack of follow-up studies.

### MARPOL annex II and EU nature directives

At present, the regulations in MARPOL Annex II allow discharges of cargo residues of hazardous non-petroleum substances, below specified volume limits, from chemical tankers during tank cleaning more than 12 nautical miles from land, including in marine protected Natura 2000 sites.

At the same time, EU member states have an obligation, due to the Habitats and Birds Directives, to protect species and habitats by designating Natura 2000 sites in both the territorial sea and in sea areas more than 12 nautical miles from land in the EEZ. Conservation plans, which define the conservation goals and conservation measures for each Natura 2000 site must also be established. The conservation plans for the Natura 2000 sites that were affected by discharges during the current study have all highlighted that discharges of chemicals pose a threat to the protected species and habitats.

Article 6(3) of the Habitats Directive, and Article 7 that extends the scope of Article 6(3) to the Birds Directive, state the following:

‘*Any plan or project not directly connected with or necessary to the management of the site but likely to have a significant effect thereon, either individually or in combination with other plans or projects, shall be subject to appropriate assessment of its implications for the site in view of the site’s conservation objectives. In the light of the conclusions of the assessment of the implications for the site and subject to the provisions of paragraph 4, the competent national authorities shall agree to the plan or project only after having ascertained that it will not adversely affect the integrity of the site concerned and, if appropriate, after having obtained the opinion of the general public*.’ (European Commission 2019).

We argue, with reference to Article 6(3), that discharges of tank washing residues from chemical tankers in Natura 2000 sites, given their potential to negatively affect populations of marine birds and other marine species and habitats for which the sites were designated, must reasonably be regarded as “likely to have significant effect” on the Natura 2000 sites in question. However, to our knowledge, no assessments have hitherto been performed by competent national authorities in Sweden or in the Baltic Sea region of the implications of discharging tank washing residues in Natura 2000 sites. Likewise, competent national authorities have never agreed to tank washing operations in Natura 2000 sites after having ascertained that they will not adversely affect the integrity of the sites concerned, that is, approved that tank washing operations are in compliance with Article 6(3) and 7 of the Habitat and Birds Directives. We therefore conclude that the present situation with recurrent discharges of hazardous non-petroleum substances in Natura 2000 sites situated more than 12 nautical miles from land, are not in line with the EU nature legislation, notably the Habitats and Birds Directives.

### Future needs

To reduce the number of legal and illegal discharges of harmful chemicals from chemical tankers the national and international regulations must be strengthened. The chemical industries, that is, not only the shipping companies, but also the exporters and importers of the transported cargo, must also immediately take responsibility for the full logistic chain and ensure that their chemical raw material and products never end up in the marine environment. Until it has been decided mandatory, the chemical industries should secure that a high quality tank washing procedure always is performed at the port of unloading, and the residues are discharged to reception facility, when ships have transported any harmful chemical in the Y category. There is also a need to improve the effectiveness of cargo tank stripping, tank washing operations and prewash procedures, especially for tall oil and similar substances with high viscosity. The possibilities to load slightly different substances on top, and thus avoiding the need for tank washing should be evaluated. From a legal and a supervisory authority’s perspective there is also a need to develop an internationally recognized method to estimate discharged volumes of various non-petroleum substances by remote sensing techniques. All types of discharges of non-petroleum substances from chemical tankers in marine Natura 2000 sites should urgently be banned.

## Conclusion

Large numbers of legal and illegal discharges of non-petroleum substances from chemical tankers were detected annually in the Baltic Sea, including in marine protected Natura 2000 sites, and the trend is increasing. Discharges of vegetable oils, other bio-based oils and biofuels may be as dangerous as discharges of petroleum oils for many marine organisms. We conclude that the regulations in the IMO MARPOL convention Annex II are not strict enough to meet the objectives of EU nature legislation regarding protection of sensitive marine species and habitats.
